# Antibiotics *versus* placebo in adults with CT-confirmed uncomplicated acute appendicitis (APPAC III): randomized double-blind superiority trial

**DOI:** 10.1093/bjs/znac086

**Published:** 2022-04-06

**Authors:** Paulina Salminen, Suvi Sippola, Jussi Haijanen, Pia Nordström, Tuomo Rantanen, Tero Rautio, Ville Sallinen, Eliisa Löyttyniemi, Saija Hurme, Ville Tammilehto, Johanna Laukkarinen, Heini Savolainen, Sanna Meriläinen, Ari Leppäniemi, Juha Grönroos

**Affiliations:** Division of Digestive Surgery and Urology, Turku University Hospital, Turku, Finland; Department of Surgery, University of Turku, Turku, Finland; Division of Digestive Surgery and Urology, Turku University Hospital, Turku, Finland; Department of Surgery, University of Turku, Turku, Finland; Department of Surgery, Jyväskylä Central Hospital, Jyväskylä, Finland; Division of Digestive Surgery and Urology, Turku University Hospital, Turku, Finland; Department of Surgery, University of Turku, Turku, Finland; Department of Gastroenterology and Alimentary Tract Surgery, Tampere University Hospital, Tampere, Finland; Faculty of Medicine and Health Technology, University of Tampere, Tampere, Finland; Department of Surgery, Kuopio University Hospital, Kuopio, Finland; Department of Surgery, Institute of Clinical Medicine, University of Eastern Finland, Kuopio, Finland; Department of Surgery, Oulu University Hospital, Oulu, Finland; Medical Research Centre Oulu, University of Oulu, Oulu, Finland; Gastroenterological Surgery, Helsinki University Hospital and University of Helsinki, Helsinki, Finland; Department of Biostatistics, University of Turku, Turku, Finland; Department of Biostatistics, University of Turku, Turku, Finland; Department of Radiology, Turku University Hospital, Turku, Finland; Department of Gastroenterology and Alimentary Tract Surgery, Tampere University Hospital, Tampere, Finland; Faculty of Medicine and Health Technology, University of Tampere, Tampere, Finland; Department of Surgery, Kuopio University Hospital, Kuopio, Finland; Department of Surgery, Institute of Clinical Medicine, University of Eastern Finland, Kuopio, Finland; Department of Surgery, Oulu University Hospital, Oulu, Finland; Medical Research Centre Oulu, University of Oulu, Oulu, Finland; Gastroenterological Surgery, Helsinki University Hospital and University of Helsinki, Helsinki, Finland; Division of Digestive Surgery and Urology, Turku University Hospital, Turku, Finland; Department of Surgery, University of Turku, Turku, Finland

## Abstract

**Background:**

Non-operative management of uncomplicated acute appendicitis is an option, but omission of antibiotics from the regimen has not been tested.

**Methods:**

A double-blind, placebo-controlled, superiority RCT in adults with CT-confirmed uncomplicated acute appendicitis was designed to compare placebo with antibiotics (intravenous ertapenem followed by oral levofloxacin and metronidazole). The primary endpoint was treatment success (resolution resulting in discharge without appendicectomy within 10 days); secondary outcomes included pain scores, complications, hospital stay, and return to work.

**Results:**

From May 2017 to September 2020, 72 patients with a mean(s.d.) age of 37.5 (11.1) years were recruited at five hospitals. Six were excluded after randomization (5 early consent withdrawals, 1 randomization protocol violation), 35 were assigned to receive antibiotics, and 31 to receive placebo. Enrolment challenges (including hospital pharmacy resources in an acute-care surgery setting) meant that only the lowest sample size of three predefined scenarios was achieved. The 10-day treatment success rate was 87 (95 per cent c.i. 75 to 99) per cent for placebo and 97 (92 to 100) per cent for antibiotics. This clinical difference of 10 (90 per cent c.i. −0.9 to 21) per cent was not statistically different for the primary outcome (1-sided *P* = 0.142), and secondary outcomes were similar.

**Conclusion:**

The lack of antibiotic superiority statistically suggests that a non-inferiority trial against placebo is warranted in adults with CT-confirmed mild appendicitis. Registration number: EudraCT 2015-003634-26 (https://eudract.ema.europa.eu/eudract-web/index.faces), NCT03234296 (http://www.clinicaltrials.gov).

## Teaser

Recent studies have suggested that supportive care alone may also be effective as a treatment option for CT-confirmed uncomplicated acute appendicitis. In this double-blind, placebo-controlled, superiority, randomized trial (APPAC III), which compared antibiotics (intravenous ertapenem followed by oral levofloxacin and metronidazole) with placebo in patients with CT-confirmed uncomplicated acute appendicitis, 66 of 72 patients completed randomized treatment with 10 days of follow-up. The 10-day treatment success rate was 97.1 (95 per cent c.i. 92.5 to 100) per cent for patients treated with antibiotics and 87.1 (77.2 to 97.0) per cent for those who received the placebo.

## Introduction

Over the past decade there has been both considerable discussion and accumulating robust evidence in both adults and children for the efficacy and safety of antibiotics as a feasible treatment alternative for imaging-confirmed uncomplicated acute appendicitis^1–6^. During the COVID-19 pandemic, antibiotics have been acknowledged as a safe alternative to surgery for uncomplicated acute appendicitis by the American College of Surgeons^7^ as non-operative treatment would allow limiting inpatient bed use and reallocation of healthcare resources.

The dogma of appendicitis inevitably progressing to perforation (without surgery) applies to complicated cases (such as obstruction with a faecolith), for which appendicectomy is indicated (unless an abscess is present^8^)^9–11^. However, the majority of patients present with imaging-confirmed uncomplicated appendicitis, in whom non-operative management with antibiotics is possible^1,3,5^. The APPAC III (APPendicitis ACuta III) trial of antibiotics *versus* placebo in adults with CT-confirmed uncomplicated acute appendicitis is a research continuum from an initial trial^3^ comparing antibiotics with appendicectomy and the recent one^5^ comparing oral antibiotics with intravenous then oral antibiotics. The trial design was based on the notion that acute appendicitis seems very similar to diverticulitis, for which uncomplicated disease does require antibiotics^12,13^. With the increasing risk of developing antibiotic resistance, studies on their rational use are warranted. A Korean single-blind randomized trial^14^ reported no difference in treatment failure rates between antibiotic use and supportive care for uncomplicated acute appendicitis. The present double-blind RCT evaluated the effect of omission of antibiotic therapy on resolution of appendicitis.

## Methods

### Study design and participants

The trial design has been described previously^15^, and the protocol including the statistical analysis plan and a full list of inclusion and exclusion criteria is available in *Appendix S1*. All patients gave written informed consent to participate. The trial was approved by the ethics committee of the Hospital District of Southwest Finland, the Finnish Medicines Agency, and by institutional boards at each participating hospital. The trial was performed in accordance with the Declaration of Helsinki. An independent data and safety monitoring committee provided trial oversight, including a preplanned interim analysis. Data were collected by the site investigators and transmitted electronically to a data coordinating centre, which kept all data confidential and blinded during the trial.

Owing to the double-blind study design, trial enrolment required use of the hospital pharmacy as the intravenous drugs could not be prepared in advance. To ensure hospital pharmacy manufacturing and drug delivery in real-life clinical practice, a pilot study with five patients was conducted between May and June 2017 after trial registration. As no changes were made to the study protocol or hospital pharmacy practice, the patients in this pilot trial were included in the study population.

Patients aged 18–60 years admitted to an emergency department with a clinical suspicion of acute appendicitis and uncomplicated acute appendicitis confirmed by CT were evaluated for enrolment in the study. Based on the study hospital CT device, contrast-enhanced CT was performed according to the study protocol recommendation either using the standard 120-kV protocol (in patients with a BMI of or over 30 kg/m^2^), the optimized 100-kV low-dose protocol (BMI less than 30 kg/m^2^) or using CT with tube current modulation (Tampere). Uncomplicated acute appendicitis was defined by an appendiceal diameter over 6 mm with a thickened, contrast-enhanced wall along with periappendiceal oedema and/or minor fluid collection and the absence of the criteria for complicated acute appendicitis (presence of appendicolith, perforation, abscess, or suspicion of tumour).

### Randomization and masking

The APPAC III trial was undertaken at four study hospitals (Turku, Tampere, Oulu, and Kuopio), in close conjunction with the concurrent APPAC II trial^5^, both focusing on the non-operative treatment of CT-confirmed uncomplicated acute appendicitis. Helsinki participated only in the APPAC III trial. In accordance with the study protocol, patients were enrolled by senior research surgeons during varying and limited hospital pharmacy office hours; Turku University Hospital had the longest availability. On admission to the emergency department during available hospital pharmacy hours, patients were first informed of the APPAC III trial, with the suggestion to participate. The concurrent APPAC II trial, with identical inclusion criteria, enrolled patients up to November 2018; all 23 patients who declined to participate in APPAC III were then offered the option to participate in APPAC II in these four study hospitals.

All patients with acute appendicitis on CT who were evaluated for enrolment in either of these two RCTs were recorded in the database; the aim was also to record all patients undergoing CT for suspected appendicitis. These evaluated patients with confirmed or suspected appendicitis were mainly reported within the APPAC II trial^5^. Owing to the variability in hospital pharmacy availability, the population of patients excluded from this trial between November 2018 and September 2019 was assessed only at the main research centre (Turku), which enrolled the majority of the patients.

Participants were randomly assigned in a 1 : 1 equal allocation ratio with random permuted blocks, and randomization was stratified by centre. The investigators were blinded to the allocation, and the randomization list was available only to the safety statistician and hospital pharmacies. The hospital pharmacy at each participating centre manufactured the trial drugs and placebo according to identical instructions. The intravenous solutions were administered in similar intravenous bags and the coloured oral capsules had an identical appearance with no differences in smell or taste; all drugs were manufactured with identical labelling.

### Treatments

Patients randomly assigned to the antibiotic group received intravenous ertapenem 1 g daily for 3 days followed by oral levofloxacin 500 mg daily and metronidazole 500 mg three times daily for 4 days. The antibiotic regimen was selected based on the previous proven efficacy in the initial APPAC trial^3^. The patients assigned randomly to the placebo group received an identical regimen of administration and treatment duration both intravenously and orally. To ensure patient safety, the minimum hospital stay was the 3-day duration of the intravenous treatment. Pain medication was prescribed according to standard hospital protocol. Pain or change in pain scores (measured on a visual analogue scale, VAS), leucocyte count, C-reactive protein (CRP) level, and temperature were recorded daily. Where there was suspicion of a lack of response to the treatment administered based on clinical findings, the patient underwent laparoscopic appendicectomy depending on the research surgeon’s decision and the reasons for proceeding to appendicectomy were recorded. Surgical findings and histopathological examination of the removed appendix were used to confirm the diagnosis; appendicitis was defined by transmural neutrophil invasion involving the appendiceal muscularis layer. Clinical diagnoses were assessed in a blinded manner by two investigators unaware of the other’s evaluation. In the event of disagreement, the clinical diagnosis was reviewed by a third investigator.

### Outcomes and measures

The primary outcome was 10-day treatment success, defined as resolution of appendicitis resulting in discharge from the hospital without appendicectomy during the follow-up. The predefined secondary endpoints at 10 days included postintervention complications related to antibiotics or placebo (all adverse events or symptoms related to either non-operative treatment of grade I or higher^16^) or appendicectomy, abdominal symptoms, duration of hospital stay, pain scores, and duration of sick leave. Cost and quality of life are not reported here.

Based on the actual clinical diagnosis, the failure rate of the randomized treatments at 10 days is reported based the primary outcome definition of treatment failure (all patients undergoing appendicectomy), along with the true primary failure rate (patients presenting with complicated acute appendicitis at surgery, that is true non-responders). After discharge, the patients were contacted by telephone at both 2–4 and 10 days. Leucocyte count and CRP levels were recorded at both 2–4 and 10 days.

### Statistical analysis

The sample size calculations required three different predefined scenarios to be created for study power analysis^15^ (*Appendix S2*). These were based on the anticipated challenges related to performing randomized trials in the emergency surgery setting, with additional obstacles created by double-blinding dependence on hospital pharmacy hours, and randomization by senior surgeon researchers. Sample size calculations were based on one-sided Pearson’s χ^2^ test for two proportions. A one-sided test was used as the hypothesis was that antibiotic treatment is superior to placebo. The sample size was calculated from an estimated success rate of 94 per cent during the hospital stay in the antibiotic group^3^. A power of 0.8 (1 − β) and one-sided significance level (α) of 0.05 were used in calculations.

Because of known challenges in the enrolment, three different target differences were used in the calculations. In scenario A, a target difference of 15 percentage points in success rate was used, leading to an estimated 79 per cent success rate in the placebo group. It was calculated that, to detect a 15-percentage point difference between antibiotic and placebo groups, 64 patients per group would be needed. With an estimated drop-out rate of 10 per cent, a total of 142 patients, 71 per group, would be enrolled in the study. In scenario B, the target difference was 20 percentage points, the estimated success rate in the placebo group was 74 per cent, and 41 patients per group would be needed. Allowing for a 10 per cent drop-out rate, a total of 92 patients, 46 per group, would be needed. In scenario C, the target difference was 25 percentage points, the estimated success rate in the placebo group was 69 per cent, and a total of 58 patients, 29 patients per group, would be needed to detect this difference. With an estimated drop-out rate of 10 per cent, a total of 64 patients, 32 per group, would be needed.

Based on predefined patient enrolment limits and timelines (if patient enrolment had not reached scenario C on 1 June 2019, scenario C with 64 patients would be the new target), scenario C was selected. The targeted minimum sample size per study hospital was set at 10 patients. Sample size calculations were performed using the Power procedure in SAS^®^ for Windows^®^, version 9.4 (SAS Institute, Cary, North Carolina, USA).

The main analyses were based on the intention-to-treat principle (all randomized patients, except for exclusion of both erroneously randomized patients with complicated appendicitis at CT and patients with early withdrawal of consent). Continuous variables are summarized as mean(s.d.) when normally distributed, and median (i.q.r.) otherwise. Categorical variables are presented as counts and frequencies. The difference in treatment success up to 10 days was tested using one-sided Fisher’s exact test. In addition, the two-sided 90 per cent confidence interval for the difference in proportions was calculated to estimate the treatment difference. Duration of sick leave was compared using two-sample *t* test and duration of hospital stay by means of the Mann–Whitney *U* test. VAS scores, leucocyte count, and CRP level (at baseline in the emergency room, at 2–4 days, and at 10-day follow-up) were analysed using linear mixed models for repeated measures. Day was considered as within factor and treatment group as between factor. Time points by group interaction described whether mean changes over time differed between groups. Study centre was handled as a random effect. Co-variance structure of unstructured was used. Sensitivity analyses were performed with Friedman test separately for each group.

All analyses were prespecified before database lock and opening of the treatment code, except for duration of symptoms before admission. There were no missing data in the primary comparison. Subjects with missing values were automatically excluded from secondary analyses. The significance level was set at 0.05 (2-tailed) and 95 per cent confidence intervals were calculated unless stated otherwise. The last day of follow-up was 25 September 2020. Data were analysed using SAS^®^ for Windows^®^, version 9.4.

## Results

### Population

**Fig. 1:**
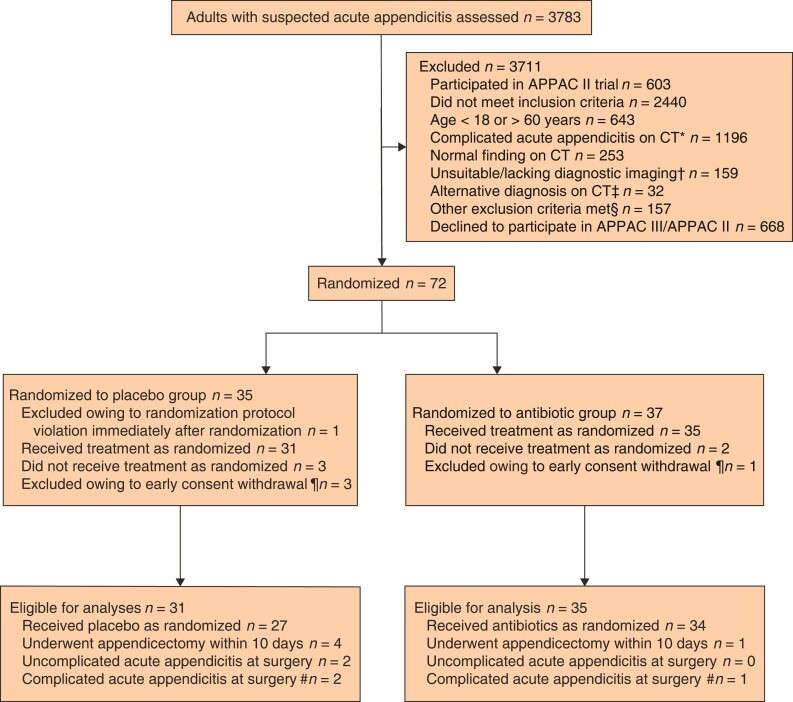
Flow chart for APPAC III trial A total of 71 patients were eligible for the baseline comparison, and 66 for comparison of the primary endpoint. *Includes appendicolith, perforation, abscess, or suspicion of tumour. †Underwent ultrasound imaging, MRI or non-contrast-enhanced CT. ‡Diverticulitis (3 patients), ovarian cyst (7), hernia (2), pelvic inflammatory disease (2), other diagnosis (18). §Pregnancy, lactation, allergy to contrast media, kidney insufficiency, use of metformin, systemic illness, and inability to provide consent. ¶Withdrew consent after randomization but before receiving randomized treatment. #Operative or histopathological findings of appendicolith, gangrene, perforation, abscess, or tumour.

From 1 May 2017 to 21 September 2020, 72 patients were assigned randomly to receive either placebo or antibiotic treatment (*Fig. 1*). One patient with complicated acute appendicitis was randomized in error and immediately excluded. After randomization, five additional patients were excluded owing to early consent withdrawal, leaving 66 patients in the primary outcome analyses (35 in antibiotic group and 31 in placebo group); all patients were available for the primary endpoint follow-up. Baseline characteristics were similar in the two groups (*Table 1*). The mean(s.d.) age was 37.5(11.1) years, and 39 per cent of the patients were women. Comparisons of baseline patient demographics between randomized and non-randomized eligible patients at Turku University Hospital, and between APPAC III and APPAC II trial patients, are presented in *Tables S1* and *S2*.

**Table 1:** Baseline characteristics

	Placebo group (*n* = 34)	Antibiotic group (*n* = 37)
**Sex**		
F	13 (38)	15 (41)
M	21 (62)	22 (60)
Age (years)*	39 (19–58)	34 (20–59)
**BMI (kg/m^2^)**†	28.1 (6.7)	28.0 (5.3)
**Body temperature (°C)**†	37.2 (0.7)	37.0 (0.5)
**VAS score for pain on admission**†	4.6 (2.8)	4.9 (2.7)
**Leucocyte count (×10^9^/l)**‡	10.5 (8.5–13.7)	11.6 (9.4–14.1)
**Neutrophil count (×10^9^/l)**‡	7.7 (5.0–10.9)	7.7 (5.5–11.4)
**C-reactive protein (mg/l)**‡	27.5 (10.2–53.0)	23.0 (11.0–45.0)
**Appendiceal diameter on CT (mm)**†	10.4 (2.7)	10.6 (2.3)
**Duration of symptoms on admission (h)**‡	19 (13–30) (*n* = 30)	14 (10–30) (*n* = 34)
**Study hospital**		
Turku	18	19
Tampere	7	8
Kuopio	3	5
Oulu	4	3
Helsinki	2	2

Baseline characteristics are shown for all randomized patients including early withdrawals, but excluding one patient with complicated acute appendicitis revealed by CT on admission, who was randomized erroneously. Values in parentheses are percentages unless indicated otherwise; values are *median (range), †mean(s.d.), and ‡median (i.q.r.). VAS, visual analogue scale.

### Primary and secondary clinical outcomes

The treatment success rate at 10 days in the placebo group was 87 (95 per cent c.i. 75 to 99) per cent, with 27 of 31 patients experiencing resolution of uncomplicated acute appendicitis and not requiring appendicectomy during the 10-day follow-up. In the antibiotics group, the treatment success rate was 97 (92 to 100) per cent as uncomplicated acute appendicitis resolved in 34 of 35 patients, with no need for appendicectomy by 10 days. Antibiotics were not statistically superior to placebo in the treatment of uncomplicated acute appendicitis; the difference between groups in treatment success was 10 (90 per cent c.i. –0.9 to 21) per cent (*P* = 0.142).

Of the five patients who underwent appendectomy during the 10 days, three presented with complicated acute appendicitis at surgery (2 after placebo and 1 after antibiotics) (*Table S3*), resulting in true primary failure (complicated acute appendicitis at surgery during primary hospitalization) rates of 7 per cent after placebo and 3 per cent after antibiotics, with a difference of 4 (95 per cent c.i. –7 to 14) per cent between the groups. The median time to appendicectomy was 6 (range 1–10) days.

The secondary outcomes with corresponding follow-up rates are summarized in *Table 2*. By 10 days, there had been no deaths and VAS pain scores decreased significantly from admission to both 2–4 and 10 days in both groups (*P* < 0.001). Adverse events and miscellaneous symptoms are summarized in *Table 3*. There were no complications related to appendicectomy within the 10-day follow-up.

**Table 2:** Secondary outcomes

	Placebo group (*n* = 31)	Antibiotic group (*n* = 35)	*P*¶
Duration of primary hospital stay (h)*	52.0 (48.3–57.4) (*n* = 31)	55.5 (50.0–59.0) (*n* = 35)	0.161#
Duration of overall hospital stay within 10 days (h)*	52.6 (48.5–61.8) (*n* = 31)	55.5 (50.0–59.0) (*n* = 35)	0.480#
**Leucocyte count (×10^9^/l)**†			Group 0.898 Day < 0.001 Group × day 0.457
Baseline	11.1 (9.9, 12.3) (*n* = 31)	11.6 (10.5, 12.8) (*n* = 35)	
2–4 days	6.7 (6.1, 7.4) (*n* = 31)	6.3 (5.7, 6.9) (*n* = 35)	
10 days	7.0 (6.1, 7.9) (*n* = 19)	6.9 (6.2, 7.5) (*n* = 18)	
**C-reactive protein (mg/l)**†§			Group 0.659 Day < 0.001 Group × day 0.684
Baseline	24.5 (16.4, 36.6) (*n* = 31)	20.1 (14.2, 30.0) (*n* = 35)	
2–4 days	22.2 (14.9, 30.0) (*n* = 30)	22.2 (16.4, 30.0) (*n* = 35)	
10 days	3.3 (1.8, 5.5) (*n* = 18)	2.6 (1.5, 4.5) (*n* = 18)	
**VAS score for pain**†			Group 0.998 Day < 0.001 Group × day 0.164
Baseline	4.5 (3.4, 5.6) (*n* = 28)	5.0 (4.0, 6.0) (*n* = 34)	
2–4 days	0.7 (0.3, 1.0) (*n* = 31)	0.2 (-0.1, 0.6) (*n* = 31)	
10 days	0.3 (0.1, 0.6) (*n* = 30)	0.3 (0.04, 0.5 (*n* = 32)	
**Duration of sick leave (days)**‡	4.7 (3.6, 5.8) (*n* = 30)	5.3 (4.2, 6.4) (*n* = 30)	0.478**

Values are *median (i.q.r.), †model-based mean (95 per cent c.i.), and ‡mean (95 per cent c.i.). §Logarithmic transformation used in analyses; estimates are transformed back to original scale. VAS, visual analogue scale. ¶*P* values for effects of linear mixed model for repeated measures (main effects of group and day, and interaction of group and day), except #Mann–Whitney *U* test and **two-sample *t* test.

**Table 3:** Adverse events related to placebo and antibiotic treatment

	Placebo group (*n* = 31)	Antibiotic group (*n* = 35)
**Related to randomized treatment**		
Skin eczema		
At 2–4 days	0	0
At 10 days	0	1
Tendinitis		
At 2–4 days	0	1
At 10 days	0	1
Candidiasis		
At 2–4 days	0	0
At 10 days	0	1
Abdominal pain		
At 2–4 days	1	1
At 10 days	0	0
**Related to possible operative treatment at 10 days**		
Abdominal or incisional pain	0	0
Surgical-site infection	0	0
**Patients with at least one adverse event**	1 (3)	4 (11)
**Other miscellaneous symptoms related to randomized treatment**		
Diarrhoea		
At 2–4 days	1	7
At 10 days	0	5
Metallic taste sensation		
At 2–4 days	0	3
At 10 days	0	0
Fatigue		
At 2–4 days	5	9
At 10 days	0	1

Values in parentheses are percentages.

## Discussion

Based on enrolment challenges of the double-blinding requiring hospital pharmacy resources in an acute-care surgery setting, the trial ended up with the smallest predefined patient number scenario—a much larger target difference between the groups than a clinically important one. As antibiotics were not superior to placebo, with a point estimate for difference of 10 percentage points between the groups, this small sample size suggests that a non-inferiority trial of supportive therapy *versus* antibiotics is warranted.

This report has limitations. Because of the anticipated challenges with patient enrolment related to dependency on the very limited hospital pharmacy hours required by the double-blinding, trial enrolment was quite slow and the authors had to settle for the predefined scenario C with the smallest number of patients. The small number of patients testing the clinically too high 25-percentage point difference can be considered a major limitation of the study, making it underpowered to draw firm conclusions. The small patient numbers lacking sufficient statistical power make the study results susceptible to type II error; even a few more patients could have had an impact on the outcomes—but this effect could naturally go both ways. However, as the only double-blind RCT comparing placebo with antibiotics, this study provides further evidence and direction for future trials as antibiotics were not superior to placebo and these promising pilot study results on symptomatic therapy need to be confirmed by a large non-inferiority RCT in the future. In fact, if this study had shown the superiority of antibiotics, taking into consideration the target difference of 25 percentage points and quite small sample size, no further studies would be needed as using a placebo would be unethical.

The limitation of enrolment being possible only during regular office hours also resulted in a lower percentage of eligible patients undergoing randomization. However, the randomized patients in this trial were quite similar to those in the concurrent APPAC II trial. There was significant variation in the number of patients recruited per study hospital, and the limited hospital pharmacy hours also had some effect on this variation resulting in the target minimum sample size per study hospital not being met. However, study hospital effect was taken into account in the analyses as a random effect.

Acute appendicitis seems to be quite similar clinically to acute diverticulitis in many respects concerning differences between the uncomplicated and complicated forms of the disease^3,8,12,17^. This resemblance has also been shown in epidemiological studies, suggesting a common underlying pathogenesis^18^. Antibiotics have shown no benefit in the treatment of uncomplicated acute diverticulitis^12,13,19,20^, and even outpatient treatment with only symptomatic care has been shown to be feasible and safe^19,21^. A double-blind design is imperative in trials assessing different drugs or the effect of a medication to minimize bias by reducing the potential for a treatment effect in patients, and the risk of researchers reporting greater effects in the treatment group or lesser effects in the placebo control group. Both the placebo effect of beneficial outcomes and the nocebo effect of negative expectations and outcomes affect the treatment response in clinical practice and clinical trials^22,23^.

With the collective substantial body of evidence on antibiotic treatment for uncomplicated acute appendicitis from large trials^1–3^, the focus of studies on non-operative treatment of uncomplicated acute appendicitis has shifted from the effectiveness of the treatment to further defining tailored optimal treatment strategies^24^. The results of these large trials^1–3^ are remarkably consistent, with a success rate of approximately 70 per cent and less disability with antibiotic therapy for uncomplicated acute appendicitis. Compared with appendicectomy, antibiotics are understandably inferior for uncomplicated acute appendicitis when evaluated only in terms of treatment efficacy^3,25,26^, as non-operative management can never compete with surgery considering the definitive nature of appendicectomy.

Potential preinterventional findings associated with a more complicated course of appendicitis, primary antibiotic treatment failure, and recurrence of appendicitis need to be identified, and international collaboration is needed to refine the diagnosis of appendicitis severity using uniform and standardized criteria. In a recent subgroup analysis of the APPAC II trial^27^, which assessed 583 patients with CT-confirmed uncomplicated acute appendicitis without an appendicolith, an appendiceal diameter of 15 mm or more and body temperature above 38°C were associated with an increased risk of non-responsiveness to initial antibiotics. The pragmatic CODA trial^1^, which included patients with more severe appendicitis, reported both a higher risk of both appendicectomy (41 per cent for patients with appendicolith and 25 per cent for patients without) and complications (12 and 4 per cent respectively). In addition to the presence of an appendicolith, similarly to APPAC II trial, the CODA trial^28^ found wider appendiceal diameter to be associated with an increased risk of appendicectomy after initial antibiotic treatment. These findings confirmed the results of earlier trials^11^ and suggest that appendicolith is a defining feature of complicated appendicitis^1^. These definitions will help clinician–patient decision-making and future trial design to clarify optimal antibiotic use or avoidance.

## Funding

This study received funding from the Sigrid Jusélius Foundation, the Academy of Finland, and the Mary and Georg C. Ehrnrooth Foundation (to P.S.). The funders of the study had no role in study design, data collection, data analysis, data interpretation, or writing of this report.

## Supplementary Material

znac086_Supplementary_Data

## Data Availability

If interested in the study data and individual-patient data, please contact the corresponding author and the data will be shared with bona fide researchers with research proposals after research completion.
