# Supplementary material for: Antibiotics versus placebo in adults with CT-confirmed uncomplicated acute appendicitis (APPAC III): randomized double-blind superiority trial
Source: Br J Surg. 2022 Apr 6;109(6):503–9. doi: 10.1093/bjs/znac086 (PMC10364767; doi:10.1093/bjs/znac086)
Supplement: znac086_Supplementary_Data [file znac086_supplementary_data.zip › Supplementary_Appendix_2.docx]

**Appendix S2. Scenarios for patient enrollment**

Sample size was calculated from an estimated success rate of 94% during hospitalization in the antibiotic group. A power of 0·8 and one-sided significance level were used in the calculations. Based on previously recognized challenges with patient enrollment in a real-life emergency setting, the hospital pharmacy hours and senior surgeons completing the randomization, the following scenarios for patient enrollment were created. Based on the predefined criteria outline in the study protocol, scenario C was selected.

|  | **Scenario A** | **Scenario B** | **Scenario C** |
| --- | --- | --- | --- |
| Clinically important difference between treatments* | 15% | 20% | 25% |
| Estimated success rate in placebo group | 79% | 75% | 69% |
| Patients per group | 64 | 41 | 29 |
| Total number of patients (including anticipated 10% dropout) | 142 | 92 | 64 |

* The estimated clinically important difference between the treatment groups or rescue appendectomy rate was determined arbitrarily based on clinical relevance at the time of study planning.
