# Supplementary material for: Antibiotics versus placebo in adults with CT-confirmed uncomplicated acute appendicitis (APPAC III): randomized double-blind superiority trial
Source: Br J Surg. 2022 Apr 6;109(6):503–9. doi: 10.1093/bjs/znac086 (PMC10364767; doi:10.1093/bjs/znac086)
Supplement: znac086_Supplementary_Data [file znac086_supplementary_data.zip › Supplementary_Tables.docx]

Table S1. APPAC III patients vs. Eligible non-randomized patients from TYKS

|  | APPAC III baseline population n=37 | Eligible non-randomized population n=385 |
| --- | --- | --- |
| Sex, n (%)  Women  Mend | 16 (43%)  21 (57%) | 211 (55%)  173 (45%) |
| Age, median (min, max) y | 38.0 (20.0-58.0) | 34.0 (18.0-60.0) |
| BMI, median (SD)* | 27.0 (5.9) | 26.3 (5.5) |
| Body temperature, mean (SD) C | 37.1 (0.5) | 37.3 (0.6) (380/385) |
| Visual analogue scale score for pain on admission, mean (SD)* | 3.7 (2.4) | 5.6 (2.4) |
| Leukocyte count, median (IQR) | 10.5 (8.4-13.7) | 12.3 (9.4-14.9) (384/385) |
| Neutrophil count, median (IQR) | 7.2 (3.9-9.5) | 9.3 (7.3-11.6) (123/385) |
| C-reactive protein, median (IQR) | 27.0 (13.0-54.0) | 32.0 (11.0-62.0) (366/385) |
| Appendiceal diameter on CT, mean (SD) mm | 10.6 (2.3) | 10.6 (2.7) |

Table S2. APPAC III patients vs. APPAC II patients

|  | APPAC III baseline population n=71 | APPAC II baseline population n=599 |
| --- | --- | --- |
| Sex, n (%)  Women  Men | 28 (39.4%)  43 (60.6%) | 263 (43.9%)  336 (56.1%) |
| Age, median (min, max) y | 36.0 (19.0-59.0) | 34.0 (18.0-59.0) |
| BMI, median (SD)* | 26.9 (6.0) (67/71) | 26.6 (5.3) (585/599) |
| Body temperature, mean (SD) C | 37.1 (0.6) (68/71) | 37.2 (0.6) (594/599) |
| Visual analogue scale score for pain on admission, mean (SD)* | 4.8 (2.8) (67/71) | 5.2 (2.4) (574/599) |
| Leukocyte count, median (IQR) | 11.8 (8.7-13.8) | 12.2 (9.3-14.9) |
| Neutrophil count, median (IQR) | 7.7 (5.5-10.6) (50/71) | 9.64 (6.5-11.9) (500/599) |
| C-reactive protein, median (IQR) | 24.7 (10.2-52.0) | 32.0 (12.0-62.5) (591/599) |
| Appendiceal diameter on CT, mean (SD) mm | 10.5 (2.5) | 10.8 (2.5) |

Table S3. Patients operated on within 10 days of randomization

| Patient Sex/Age | Preintervention imaging | Intervention group | Days to operation after randomization | Surgical/histopathological findings | Retrospective CT analysis |
| --- | --- | --- | --- | --- | --- |
| Female/29 | Uncomplicated appendicitis, appendix diameter 12 mm | Placebo | 1 | Uncomplicated acute appendicitis |  |
| Male/54 | Uncomplicated appendicitis, appendiceal diameter 16 mm | Placebo | 2 | Gangrene, abscess, perforation | Complicated acute appendicitis, appendiceal diameter 13 mm |
| Female/46 | Uncomplicated appendicitis, appendix diameter 14 mm | Antibiotics | 1 | Gangrene | Complicated acute appendicitis, appendiceal diameter 13 mm |
| Female/39 | Uncomplicated acute appendicitis, appendix diameter 8 mm | Placebo | 7 | Uncomplicated acute appendicitis |  |
| Male/27 | Uncomplicated appendicitis, appendix diameter 10 mm | Placebo | 10 | Abscess, gangrene | Uncomplicated acute appendicitis, appendiceal diameter 13 mm |
