# Supplementary material for: Antibiotics versus placebo in adults with CT-confirmed uncomplicated acute appendicitis (APPAC III): randomized double-blind superiority trial
Source: Br J Surg. 2022 Apr 6;109(6):503–9. doi: 10.1093/bjs/znac086 (PMC10364767; doi:10.1093/bjs/znac086)
Supplement: znac086_Supplementary_Data [file znac086_supplementary_data.zip › Supplementary_Appendix_1.docx]

**Appendix S1. Protocol for ‘Antibiotics versus placebo in adults with CT-confirmed uncomplicated acute appendicitis (APPAC III): randomized double-blind superiority trial’**

Salminen Paulina, MD, PhD^1,2^, Sippola Suvi, MD^1,2,3^, Haijanen Jussi, MD^1,2^, Nordström Pia, MD, PhD^4,5^, Rantanen Tuomo, MD, PhD^6,7^, Rautio Tero, MD, PhD^8,9^, Sallinen Ville, MD, PhD^10^, Löyttyniemi Eliisa, MSc^11^, Hurme Saija, MSc^11^, Tammilehto Ville, MD^12^, Laukkarinen Johanna, MD, PhD^4,5^, Savolainen Heini, MD, PhD^6,7^, Meriläinen Sanna, MD, PhD^8,9^, Leppäniemi Ari, MD, PhD^10^, Grönroos Juha, MD, PhD^1,2^

1. Division of Digestive Surgery and Urology, Turku University Hospital, Turku, Finland
2. Department of Surgery, University of Turku, Turku, Finland
3. Department of Surgery, Jyväskylä Central Hospital, Jyväskylä, Finland
4. Department of Gastroenterology and Alimentary Tract Surgery, Tampere University Hospital
5. Faculty of Medicine and Health Technology, University of Tampere, Tampere, Finland
6. Department of Surgery, Kuopio University Hospital, Kuopio, Finland
7. Department of Surgery, Institute of Clinical Medicine, University of Eastern Finland.
8. Department of Surgery, Oulu University Hospital, Oulu, Finland
9. Medical Research Center Oulu, University of Oulu, Finland
10. Gastroenterological Surgery, Helsinki University Hospital and University of Helsinki, Helsinki, Finland
11. Department of Biostatistics, University of Turku, Turku, Finland
12. Department of Radiology, Turku University Hospital, Turku, Finland

**This supplement contains the following items:**

1. **Study protocol**
2. **Statistical analysis plan**

**APPAC III Study protocol 29.3.2018**

**Antibiotic therapy vs. placebo in the treatment of acute uncomplicated appendicitis: a prospective randomized double-blind placebo-controlled trial**

**Paulina Salminen, MD, PhD**

**Turku University Hospital**

**Table of contents** 2

1. Background 3

1.1. The APPAC trial 3

1.2. The diagnosis and treatment of acute appendicitis 5

1.2.1. Uncomplicated and complicated acute appendicitis 5

1.2.2. Computed tomography (CT) in diagnosing acute appendicitis 6

1.2.3. Treatment of acute appendicitis 7

1.3. Spontaneous resolution of acute appendicitis and acute diverticulitis 7

2. Aims of the study and study hypothesis 8

3. Combination of APPAC II and APPAC III studies in clinical practice 8

4. Combination of the APPAC III and MAPPAC studies in clinical practice 8

5. Study design, patients and methods 9

5.1. Trial design 9

5.2. Participants 9

5.3. Registration and randomization 10

5.4. Interventions 10

5.5. Outcome parameters 11

5.6. Data collection and follow-up 12

6. Statistical methods 13

6.1. Statistical hypothesis 13

6.2. Sample size calculations 13

6.3. Interim analysis 13

6.4. Statistical analysis 14

7. Ethical considerations and study relevance 14

8. Study costs 15

9. Study schedule 16

10. Study hospitals and investigators 17

11. References 17

**1. Background**

Appendectomy has unquestionably been the standard treatment for acute appendicitis for over a century. More than 300.000 appendectomies are performed annually in the United States^1^. Although appendectomy is generally well tolerated, it is a major surgical intervention and can be associated with postoperative morbidity ^2, 3^.

Since the time Fitz described the relationship between the appendix and pelvic abscess and McBurney demonstrated reduced morbidity from pelvic infections attributable to appendectomy, it has been thought that acute appendicitis invariably progresses to perforation. This line of thinking underlies the belief that emergency appendectomy is required when a diagnosis of appendicitis is made^4, 5^. Fitz and McBurney’s publications predated the availability of antibiotics by 40 years. In the absence of antibiotics, appendectomy saved lives by reducing the risk of uncontrolled pelvic infection when appendicitis was present.

Even though appendectomy has been the mainstay treatment for appendicitis, relatively soon after antibiotics were available, Coldrey reported treating 471 acute appendicitis patients with antibiotic therapy in 1959. Mortality was low (0.2 %) and recurrent appendicitis occurred only in 14.4 % of patients ^6^. More recently, the notion of treating appendicitis with antibiotics was tested in 3 randomized clinical trials (Table 1^7^) ^8-10^.Their results were summarized in a Cochrane analysis ^11^ and several meta-analyses. ^12-16^ Each of these trials had limitations and appendectomy has remained the standard approach for treating appendicitis.

- 1. **The APPAC trial**

In order to compare antibiotic therapy with appendectomy in the treatment of CT-scan confirmed uncomplicated acute appendicitis, we conducted the APPAC trial enrolling patients from November 2009 to June 2012. The APPAC trial ^7^ is a multicenter, randomized, open-label, non-inferiority trial conducted in Finland enrolling 530 patients 18 to 60 years of age with a CT scan confirmed uncomplicated acute appendicitis. Patients were randomly assigned to early appendectomy or antibiotic treatment with a follow-up of one year. Antibiotic therapy was intravenous ertapenem for three days followed by seven days of oral levofloxacin and metronidazole treatment; patients randomized to the operative treatment group underwent standard, open appendectomy.

The primary endpoint for surgical intervention was the successful completion of an appendectomy. The primary endpoint for antibiotic treated patients was discharge from the hospital without the need for surgery and no recurrent appendicitis during a follow-up of one-year.

A pre-specified non-inferiority margin of 24 percentage points for the difference between treatments was used. Secondary pre-specified endpoints included hospital stay, post-intervention pain, sick leave and overall morbidity.

There were 273 patients in the operative group and 257 in the antibiotic group. All but 1 patient in the surgery group underwent successful appendectomy resulting in a 100% (272/273; CI 98.9 – 100.0) success rate. In the antibiotic group, 70 patients (27.3 %: (CI 22.0 – 33.2) underwent surgical intervention within 1 year of initial presentation for appendicitis and 186 of 256 patients available for follow-up (72.7%; CI 66.8 – 78.0) did not require surgery. The intent-to-treat analysis yielded a success rate difference of 27.3 % (CI 22.0 – 33.2). Given our pre-specified non-inferiority margin of 24%, we were unable to demonstrate non-inferiority of antibiotic treatment relative to surgery. Of the patients randomized to antibiotic treatment, who subsequently underwent appendectomy, 58 (82.9%; CI 72.0 – 90.8) had uncomplicated appendicitis, 7 (10.0%; CI 4.1 – 19.5) had complicated appendicitis and 5 (7.1%; CI 2.4 – 15.9) operated for suspected recurrence did not have appendicitis. There were no intra-abdominal abscesses or other major complications associated with delayed appendectomy in this group.

Antibiotic treatment of patients with uncomplicated acute appendicitis was not shown to be non-inferior to appendectomy for uncomplicated appendicitis within the first year of observation following initial presentation of appendicitis. The pre-specified non-inferiority margin was established somewhat arbitrarily because little clinical information was available to make a better estimate. However, the majority (73%), of patients with uncomplicated acute appendicitis were successfully treated with antibiotics. None of the patients, who initially were treated with antibiotics that later had appendectomy, had major complications. These results suggest that CT-proven uncomplicated acute appendicitis is not a surgical emergency and antibiotic therapy is a safe first-line treatment of uncomplicated acute appendicitis. With the development of more precise diagnostic capabilities like CT and effective broad-spectrum antibiotics, appendectomy may be unnecessary for uncomplicated appendicitis, which occurs in the majority of acute appendicitis cases. Patients should be able to make an informed decision between antibiotic treatment and appendectomy and focus should also be on taking into account the patient-centric outcomes. Future studies should focus both on early identification of complicated acute appendicitis patients needing surgery and to prospectively evaluate the optimal use of antibiotic treatment in patients with uncomplicated acute appendicitis.

**1.2. The diagnosis and treatment of acute appendicitis**

Acute appendicitis is the most common cause of abdominal pain in emergency departments and appendectomy is the most common emergency abdominal surgery. The lifetime risk of acute appendicitis in males is 8.6% and 6.7% in females. ^17^ In Finland according to Stakes data there were 6 377 appendectomies (3242 in males, 3135 in females, median age 35 years) performed in 2010 with a total number of days in hospital care was 16 111 days and the mean length of hospital stay was three days.

Although acute appendicitis is the most common reason for surgical emergency department visit, its diagnosis still remains challenging. The clinical diagnosis has previously been based on patient history, physical examination and laboratory findings as well as the clinical surgical diagnosis. Several scoring systems have been created to aid in the diagnosis of acute appendicitis^18-20^, but the accuracy of clinical diagnosis without preoperative imaging is about 76 – 80 % for combined patient groups of males and females^21, 22^.

As acute appendicitis has historically been thought to always progress to perforation requiring emergency appendectomy, high negative appendectomy rates even up to 40 % in some patient populations have been previously accepted as good surgical practice. For the last two decades, the use of dedicated imaging in acute abdomen in general and also in acute appendicitis has led to improved diagnostic accuracy.

**1.2.1. Uncomplicated and complicated acute appendicitis**

Based on large epidemiological studies, we now know that complicated (perforated) and uncomplicated (non-perforated) appendicitis have followed different epidemiological trends. These unassociated epidemiologic trends suggest different pathophysiology for the two form of appendicitis. The differential diagnosis is essential as patients with an uncomplicated acute appendicitis may not require surgical intervention and might experience even spontaneous resolution without perforation.^23^ The majority (approximately 80 %) of acute appendicitis cases are of uncomplicated nature.

Complicated acute appendicitis defined as a finding of a perforation, appendicolith, abscess or a suspicion of a tumor, requires emergency appendectomy with the exception of cases with abscess as they are often managed conservatively.

Appendicolith is a calcified fecal concretion in the appendix resulting in internal luminal obstruction and it is the most common form of complicated acute appendicitis. In the first randomized study by Vons et al.^10^ comparing operative treatment and antibiotic therapy using CT as a diagnostic inclusion criterion, the presence of an appendicolith in preoperative CT scan was the only factor that significantly increased the risk of complicated appendicitis and it was also the only factor associated with the failure of antibiotic therapy for acute appendicitis. Indeed, if Vons et al^10^ had excluded the patients with an appendicolith from their analysis, no significant difference in the incidence of post-intervention peritonitis between the treatment groups would have been noticed in their study.

**1.2.2. Computed tomography (CT) in diagnosing acute appendicitis**

CT imaging is the primary imaging modality and the golden standard in the diagnosis of acute appendicitis as it establishes the diagnosis with almost perfect diagnostic accuracy. The advantages of CT imaging are high accuracy, availability, ease of performance and interpretation, and that it is rarely affected by bowel gas, severe abdominal pain or extreme body habitus. The main disadvantage of CT is exposure to radiation.

The increased use of preoperative CT imaging has been evaluated thoroughly by evaluating its impact on the negative appendectomy rate reducing the number of unnecessary appendectomies. In 2010, a mandatory imaging guideline for suspected acute appendicitis was implemented in the Netherlands. After implementation the negative appendectomy rate dropped significantly from 23 % to 6 % (p<0.001) reducing the surgical complication rate from 20% to 14 % and resulting in average cost-per-patient decrease by 594€.^24^

The favorable diagnostic performance of CT imaging has encouraged optimization of the protocol to minimize exposure to radiation through the development of low-dose CT protocols. Low-dose protocols balance with as low as reasonably achievable-principle while maintaining diagnostic accuracy. However, low-dose protocols with intravenous contrast are still not implemented in routine clinical practice. These protocols require more advanced optimization and validation because of the wider need for contrast enhanced assessment. Kim et al^25^ showed that contrast enhanced low-dose CT (median radiation dose 116mmGy in dose-length product) was not inferior to standard-dose contrast enhanced CT (median radiation dose 521 mmGy), with negative appendectomy rates of 3.5% and 3.2% respectively and no statistical significance in appendiceal perforation rates or patients requiring additional imaging.

We have initiated a prospective observational study (OPTICAP trial, NCT02533869, Ethical committee of Turku University Hospital approval) in order to optimize a low-dose CT scan for both diagnosing acute appendicitis and to differentiate uncomplicated acute appendicitis from a complicated acute appendicitis. In this study we have performed phantom imaging with 15 different imaging protocols aiming to minimize radiation with optimal diagnostic accuracy. The phantom protocols were assessed by blinded evaluation of two gastrointestinal radiologists and the two best performing protocols were chosen for the clinical phase. The clinical evaluation included performing both of these imaging protocols for patients with suspected uncomplicated acute appendicitis evaluated by a senior digestive surgeon. All of the enrolled patients underwent laparoscopic appendectomy to evaluate the sensitivity and specificity of the imaging protocols. The most optimal imaging protocol will be selected for use in the APPAC II and III trials; the final results will be available in September 2016.

**1.2.3. Treatment of acute appendicitis**

For over a century appendectomy has been the standard treatment for all patients with acute appendicitis. However, the results of our APPAC trial have now shown that the majority (73%) of patients with uncomplicated acute appendicitis were successfully treated with antibiotics alone. We also showed that none of the patients treated initially with antibiotics and later undergoing appendectomy had major complications or increased morbidity defining antibiotic therapy as a safe first-line treatment. Patients with a complicated acute appendicitis require emergency appendectomy and early identification of these patients is of vital importance. Laparoscopic appendectomy has become the golden standard for appendectomy providing lower morbidity and faster recovery compared with open appendectomy. For patients with uncomplicated acute appendicitis, the time has come to evaluate abandoning routine appendectomy and evaluating the optimal use of antibiotic therapy.

**1.3. Spontaneous resolution of acute appendicitis and acute diverticulitis**

Emergency appendectomy was first advocated because of the very high mortality of perforated appendicitis combined with the assumption of the natural course of acute appendicitis evolving always to perforated disease. This was first reported in 1886 by Reginald Fitz, who initially originated the term appendicitis and identified appendix as a cause for right lower quadrant infections. Fitz also noted that one-third of patients in a large autopsy series from the pre-appendectomy era had evidence of prior appendiceal inflammation suggesting spontaneous resolution of acute appendicitis.^4^

Acute appendicitis is similar to acute diverticulitis (“left-sided appendicitis”) and this similarity has been shown in epidemiological studies suggesting a common underlying pathogenesis.^1^ There is one multicenter randomized trial^26^, one population-based study^27^, one case-control study^28^ and one prospective observational study^29^ that have shown no benefit of antibiotic therapy in uncomplicated acute diverticulitis. The reported complication rates in these studies is low (approximately 2 %) and even outpatient management without antibiotics in acute uncomplicated diverticulitis has been shown to be feasible, well-functioning and safe^29^.

**2. Aims of the study and study hypothesis**

The aim of the study is to compare antibiotic therapy with placebo in the treatment of uncomplicated acute appendicitis to evaluate the role of antibiotic therapy in the resolution of acute uncomplicated appendicitis. The study hypothesis is that antibiotic therapy is necessary in the treatment of acute uncomplicated and that antibiotic therapy is superior to spontaneous resolution (placebo) with the primary endpoint evaluated at ten days after the intervention.

**3. Combination of APPAC II and APPAC III studies in clinical practice**

APPAC II and APPAC III trials are separate studies regarding the applied study permissions (Fimea, Tukija, the Ethical committee of Turku University Hospital). In practice these two studies will be performed in close conjunction with each other as the enrolled patient population is identical in both studies and the study chosen for enrollment will be based mainly on the time of day (based on study design APPAC III enrollment is only possible between 8 a.m. and 2 p.m.) and secondly on patient preference (if the patient is unwilling to participate in APPAC III, they will be informed and invited to participate in APPAC II trial). After 2 p.m. until 8 a.m. all of the eligible patients will be invited to participate in APPAC II trial.

**4. Combination of APPAC III and MAPPAC studies**

**MAPPAC study and** The MAPPAC trial assesses the microbiological etiology of appendicitis and the impact of antibiotic therapy on gut microbiota. MAPPAC trial will be enrolling in conjunction with the APPAC II and III trials. APPAC III and MAPPAC trials are separate studies regarding the applied study permissions (Fimea, Tukija, the Ethical Committee of Turku University Hospital). Patients recruited for the APPAC III trial will asked to sign an informed consent form allowing for the use of their data and collection of microbiological samples for the vice versa MAPPAC trial patients will be informed that MAPPAC study data will be used in conjunction with APPAC II trial.

**5. Study design, patients and methods**

**5.1. Trial design**

The trial has been designed as a prospective randomized double-blind, placebo-controlled, superiority multicenter trial to compare antibiotic therapy with placebo in the treatment of acute uncomplicated appendicitis.

**5.2. Participants**

Patients presenting with suspected acute appendicitis will be enrolled from six participating Finnish hospitals: all five university hospitals (TYKS, OYS, HYKS, TAYS, KYS) and one central hospital (Jyväskylä). The APPAC III trial participation is restricted to the university hospitals and one large central hospital based on the availability of hospital pharmacy resources, which are essential in conducting the trial in a reliable double-blinded study design. All adult patients (aged 18 – 60 years) admitted to the emergency department with a clinical suspicion of uncomplicated acute appendicitis will undergo a low-dose CT scan optimized for the diagnosis of acute appendicitis (OPTICAP trial, please see chapter 1.2.2.). Clinical history, physical investigation, VAS pain scores (visual analogue scale) and laboratory tests will be recorded for all of the evaluated patients in a prospective online database (BCB Medical APPAC-database developed by our study group). An informed consent will be obtained from all of the patients.

Inclusion and exclusion criteria for both APPAC II and III trials are identical.

Inclusion criteria: 1) Signed informed consent, 2) Age 18 – 60 years, 3) CT scan confirmed diagnosis of uncomplicated acute appendicitis.

Exclusion criteria: 1) Age <18 or > 60 years, 2) Pregnancy or lactating, 3) Allergy to contrast media or iodine, 4) Renal insufficiency, 5) Allergy or contraindication to antibiotic therapy 6) Metformine medication, 7) Severe systemic illness (for example malignancy, medical condition requiring immunosuppressant medications), 8) Complicated acute appendicitis in a CT scan (appendicolith, perforation, abscess, suspicion of a tumor), 9) Inability to co-operate and give informed consent.

Contraindications for the use of antibiotics include either allergy to the antibiotic regimen or auxiliary substance or interaction with other medications. In the case of quinolones, epilepsy and previously diagnosed tendinitis or tendon rupture related to quinolone treatment are contraindications. Other overall contraindications to antibiotic treatment in general include pregnancy, lactation, and the age under 18 years; do not apply as these patients will not be evaluated for enrollment in the study based on exclusion criteria.

According to the study protocol all patients admitted to the emergency room with suspected acute appendicitis will undergo CT imaging as CT has become the golden standard imaging in diagnosing acute appendicitis. Based on our OPTICAP-trial, the CT scan protocol used for acute appendicitis will be optimized for radiation exposure. If complicated acute appendicitis is diagnosed on CT, patients will undergo a laparoscopic appendectomy within eight hours (the patients will be classified as “requiring surgery within 0- 8 hours” in an acute care surgery criteria used in the operating theatre). In order to collect all acute appendicitis patients both to prevent any bias and to enable a thorough conception of acute appendicitis as an emergency abdominal condition, all patients with suspected acute appendicitis undergoing a CT, will be thoroughly recorded, the patients will be informed about data collection and an informed consent will be obtained. Additionally, an extra serum sample will be collected for later immunological analyses regarding patients presenting with complicated acute appendicitis (MAPPAC trial) and patients enrolled in APPAC III trial.

**5.3. Registration and randomization**

Patients will be randomized with a 1:1 equal allocation ratio to receive either antibiotic therapy or placebo. A safety statistician will perform the randomization procedure. Randomization will be made by center using random permuted blocks. The randomization and the used randomization blocks will be blinded to the investigators. The randomization listing will be made available only for safety statistician and the hospital pharmacies based on patient numbers. To ensure patient safety regarding the possible emergency opening of the double-blinded randomization code, the whole randomization list will be made available by phone call at Turku University Hospital Pharmacy at all times.

**5.4. Interventions**

The treatment arms will be antibiotic therapy vs. placebo. The antibiotic therapy is similar to the APPAC trial as we need to have antibiotic therapy with proven efficacy in order to compare the therapy with placebo. The only difference in the antibiotic therapy between APPAC and APPAC III trial is the total duration of the p.o. antibiotic therapy; in APPAC trial patients received seven days of p.o. antibiotics and in APPAC III trial this is shortened to four days. In APPAC III trial for patients randomized to antibiotic therapy, i.v. ertapenem sodium 1 g will be administered for three days with the first dose given in the acute care surgery ward. The i.v. ertapenem will be followed by p.o. levofloxacin 500 mg x 1 and metronidazole 500 mg x 3 for four days resulting in total duration of one week for the treatment. For patients randomized to placebo group, i.v. placebo and p.o. placebo will be administered according to the same protocol. None of the people involved in the treatment of patients will be aware of the patient’s treatment arm as hospital pharmacy will mask all of the i.v. packages to be of exactly of same appearance. This will also be the case for the p.o. tablets as they will be manufactured either in the hospital pharmacy or by a qualified medical company resulting in similar capsules filled with either the antibiotic regimen or placebo. To ensure patient safety in the placebo group, all of the patients will be monitored in the hospital for three days (duration of the i.v. treatment).

If the patient is suspected of not responding to the antibiotic or the placebo therapy during the primary hospitalization, the following outcome parameters (VAS/changes in VAS, leukocyte count, CRP, temperature, status findings) will be registered in the database. To ensure patient safety in cases of suspected progression of the acute appendicitis, the patient will be operated on based on the surgeon’s decision. The operative finding and the histopathology of the appendix will be recorded in the database.

After the initial hospitalization recurrent acute appendicitis will be diagnosed on a clinical basis and a patient with a suspected recurrence will undergo laparoscopic appendectomy and the recurrent acute appendicitis will be verified by histopathological examination of the removed appendix. In cases of patients undergoing appendectomy for treatment failure, we will inform the patients, that further specialized histopathological analysis may be performed in addition to standard histopathological examination.

An extra serum sample will be obtained for future immunological analyses and all enrolled APPAC III patients are informed of collecting this extra serum sample and about performing immunological and possible other analysis using this acquired extra serum sample; this information is stated in the informed consent.

**5.5. Outcome parameters**

The primary endpoint is the success of the randomized treatment (treatment efficacy). The treatment success is defined as the resolution of acute appendicitis with study treatment resulting in discharge from the hospital without the need for surgical intervention and treatment efficacy evaluated at ten days after initiation of the randomized treatment. Secondary endpoints include post-intervention complications (Clavien-Dindo classification), late recurrence of acute appendicitis after study treatment defined as clear clinical suspicion of acute appendicitis evaluated at follow-up of one, three, five and ten years, duration of hospital stay, VAS scores, quality of life (QOL, 15D), sick leave and treatment costs.

The MAPPAC study feces samples regarding APPAC III trial patients will be stored according to regulations. These samples will be used only for MAPPAC and APPAC III trials. All participating patients in each trial will be informed about combining the MAPPAC and APPAC III data.

**5.6. Data collection and follow-up**

After signed informed consent, all of the patients evaluated for acute appendicitis and study enrollment are registered to an online database at each participating institution. The researchers together with BCB Medical have created the online database, where all patients evaluated for enrollment in the study will be recorded. To ensure thorough data collection and to be able to evaluate selection bias, all of the patients presenting with acute appendicitis at the research hospitals will be recorded in the database. The information recorded from the patients who are not participating in APPAC II or APPAC III studies is used only by the regulations of register based studies. These patients not included in either of the APPAC trials will be informed of this data collection according to the guidelines of the Finnish health Ministry (STM) and their informed consent will be obtained. The data collection will be sent online to the database and Turku University Hospital as the main research center will be in charge of the common database with full access to the data. The researchers need the full access to the data in order to be able to correct possible false data entries, to enter possible missing data and to be able to keep up with the number of enrolled patients. The online database will not be used for other purposes during the trial and all of the visits to the database will be recorded in the database log. The researchers will not have access to APPAC III trial randomization groups through the database, please see 4.3.

The follow-up for patients will include laboratory tests (leukocyte count, CRP) and a phone call 2-4 days after discharge from the hospital. For most of the patients, the follow-up is aimed at three days after discharge. If the discharge form the hospital will take place on Wednesday, the patient will be evaluated on Fridays and if the discharge will be on a Thursday, the patient will be evaluated on the following Monday. All of the patients will also be evaluated at ten days after the discharge by a phone call. The follow-up by a phone call will be performed at one, three, five and ten years.

**6. Statistical methods**

**6.1. Statistical hypothesis**

The primary objective of the study is to compare the success of treatment between antibiotic therapy and placebo group within ten days after initiation of the randomized treatment. Superiority of antibiotic group vs. placebo group will be tested using following statistical hypothesis:

H_0_: p_1_ ≤ p_2_

H_1_: p_1_ > p_2_

where p_1_ is success of treatment proportion of antibiotic group and p_2_ for placebo group.

**6.2. Sample size calculations**

Sample size calculations were based on one-sided Pearson’s χ^2^ -test for two proportions. Sample size was calculated from an estimated success rate of 94% during the hospitalization in antibiotic group ^7^. A decrease of 15 percentage points in success rate is considered clinically important difference leading to estimated 79% success rate in placebo group. We estimated that to detect a 15 percentage points difference (antibiotics – placebo) between groups with a power of 0.8 (1-β) and one-sided significance level (α) of 0.05 64 patients per group is needed. Before the trial initiation, 3-5 pilot patients will be recruited in order to finalize the hospital pharmacy procedures and drug delivery in clinical practice. Based on the pilot study enrollment speed and the recognized challenges in conducting trial, in real-life emergency setting we need to take into account the anticipated enrolment delays to assure the completion of this trial within reasonable time. These challenges consisting of emergency surgery patient enrolment dependent on hospital pharmacy services available only during standard working hours, the requirement for senior surgeon enrolment and the discrepancy between the admission hours of appendicitis patients and hospital pharmacy working hours mandated us to create three scenarios for study power analysis and the number of patients to be enrolled.

In scenario A, a decrease of 15 percentage points in success rate is considered clinically important difference leading to estimated 79% success rate in placebo group. We estimated that to detect a 15-percentage points difference (antibiotics – placebo) between groups 64 patients per group is needed. With an estimated dropout rate of 10% total of 142 patients, 71 patients per group will be enrolled in the study. In scenario B, clinically important difference is 20 percentage points, estimated success rate in placebo group is 74% and to detect this difference 41 patients per group is needed. With an estimated dropout rate of 10% total of 92 patients, 46 patients per group will be enrolled in the study. In scenario C, clinically important difference is 25 percentage points, estimated success rate in placebo group is 69% and to detect this difference 29 patients per group is needed. With an estimated dropout rate of 10% total of 64 patients, 32 patients per group will be enrolled in the study. Targeted minimum sample size per study hospital will be 10 patients. One-sided test will be used as our hypothesis is that antibiotic treatment is more effective treatment than placebo. Sample size calculations were performed using Power procedure in SAS System for Windows, Version 9.4 (SAS Institute Inc., Cary, NC).

On June 1^st^, 2019, a study committee consisting of the outside safety monitoring committee and the core research group will assess which scenario will be chosen for patient enrollment with plan A being the target scenario. Active recruitment at this point will be continued until a decision is made on the clinically realistic scenario. The date of evaluation is set approximately 1 year after the last study hospital has initiated study enrollment. If the patient enrollment on June 1^st^, 2019, has reached the patient number in scenario B (92 patients), recruitment will continue until the original target scenario A (142 patients) has been reached. If scenario B enrollment has not been reached at this evaluation time point, but scenario C (64 patients) has, then scenario B will be the new target scenario. If patient enrollment has not reached scenario C by June 1^st^, 2019, scenario C will be the new target scenario.

**6.3. Interim analysis**

For the safety of the patients in the placebo treatment group an interim analysis will be conducted after 35 patients per group are enrolled to study and followed for ten days. For the interim analyses a safety statistician with independent trial safety monitoring committee will open the treatment code and calculate the point estimate of the proportion in each group. If the proportion in at least one of the groups is below 50%, the study will be terminated. The safety statistician and the independent committee members will be the only ones who know the exact proportions. The investigators and the study statistician will only be informed of the continuation or the termination of the study. No statistical tests will be conducted in interim analysis and therefore no corrections to the p-values are needed in the final analyses of study. In addition to the interim analysis, the safety monitoring committee may be asked to meet ad hoc, if evaluated necessary by the researchers. The safety monitoring committee will consist of the safety statistician Eliisa Löyttyniemi (Department of biostatistics, Turku University), professor Päivi Rautava (Chief of clinical research, Turku University Hospital), Susanna Tuomaala (Turku Clinical Research Center) and Jari Ovaska, MD, PhD (Chief of Digestive Surgery).

**6.4. Statistical analyses**

Categorical variables of the study will be characterized by treatment using frequencies and percentages and for continuous variables means and standard deviations or medians with range and 25^th^ and 75^th^ percentiles will be used. Difference in treatment success between antibiotic and placebo group will be tested using Fisher´s one-sided test. The two-sided 90% confidence interval (CI) for proportion difference will be calculated as well to estimate the treatment difference. The secondary outcomes will be analyzed using chi-squared test, independent samples t-test or Mann-Whitney U-test. The assumptions of tests will be checked for justification of the analyses. For the secondary outcomes two-sided p-values will be used. The study site differences will be evaluated in statistical models and if major differences are detected more complicated statistical models will be used in the analyses of primary and secondary outcomes. P-values less than 0.05 will be considered statistically significant. The main analyses will be based on the intention-to-treat (ITT) principle (all randomized excluding possible erroneously randomized patients with CT diagnosis of complicated appendicitis). The subjects with missing data will automatically be excluded from the analyses of the variables in concern. Statistical analyses will be performed using SAS System for Windows, Version 9.4 or later (SAS Institute Inc., Cary, NC).

**7. Ethical considerations and study relevance**

Both APPAC II and APPAC III study protocols are based on the results of our randomized APPAC trial comparing antibiotic therapy with appendectomy in the treatment of uncomplicated acute appendicitis. Based on the results of our APPAC trial, we now know that the majority (73%) of patients with uncomplicated acute appendicitis can be safely treated by antibiotics alone and that none of the patients, who initially were treated with antibiotics that later had appendectomy, had major complications. These results suggest that CT-proven uncomplicated acute appendicitis is not a surgical emergency and antibiotic therapy is a safe first-line treatment of acute uncomplicated appendicitis. The APPAC study results are based on the accurate diagnosis of acute appendicitis and CT imaging has become the golden standard in diagnosing acute appendicitis. Prior to initiation of the APPAC II and III trials, we are aiming to minimize the radiation exposure by optimizing a low-dose CT protocol combining high sensitivity and specificity with markedly reduced radiation exposure (the OPTICAP trial). The APPAC II trial will evaluate the treatment of uncomplicated acute appendicitis with two different antibiotic therapies aiming to optimize the antibiotic treatment by shortening the duration of the treatment, taking into account the antibiotic resistance problem by evaluating less broad-spectrum antibiotics and minimizing the required hospital stay.

The relevance of our previous APPAC trial has been substantial in initiating worldwide discussion and evaluation of the optimal treatment for uncomplicated acute appendicitis as the time has come to abandon routine appendectomy for uncomplicated acute appendicitis. The changes in the treatment paradigm for CT-proven uncomplicated acute appendicitis will naturally require further prospective studies, but avoiding unnecessary appendectomies will result in major cost savings and markedly decreased operative morbidity. APPAC II trial results will further enhance the thorough evaluation of the use of antibiotic therapy in uncomplicated acute appendicitis as by optimizing the antibiotic treatment will result in further cost savings and better utilization of hospital resources. As we now have the results of our initial APPAC trial, the international study focus on acute appendicitis will be the evaluation of the non-operative management and its optimization. Based on our APPAC trial, we are in the frontline of this research even from an international point of view and both APPAC II/III study hypothesis are the key questions in this research field.

Acute appendicitis is one of the most common surgical emergencies and appendectomy is the most common surgical emergency operation with approximately 300.000 annual procedures in the US and 6500 appendectomies in Finland. The results of both the completed and future APPAC trials are very likely to have a profound impact on the treatment paradigm of uncomplicated acute appendicitis by avoiding unnecessary surgeries and the related morbidity resulting in major cost savings.

**8. Study costs**

Based on our APPAC trial results, antibiotic therapy is a safe first-line treatment for uncomplicated acute appendicitis. The diagnosis of acute appendicitis with a CT can be achieved with almost perfect diagnostic accuracy and CT imaging is now considered standard in diagnosing acute appendicitis. Regarding the diagnosis required for the patient enrollment, the APPAC III trial does not deviate from the standard care for acute appendicitis and thus there are no extra costs regarding the study inclusion diagnostics. However, the APPAC III trial requires funding regarding the arrangements for adequate double-blinded randomized trial (hospital pharmacies, preparation of i.v. medications, preparation of p.o. medications); the total cost evaluation is based on the information and cost evaluation from Turku University hospital pharmacy. All of the participating hospital pharmacies require their own contracts and the prices may somewhat vary, but the estimated total costs for the first year enabling the APPAC III study initiation is approximately 45.500€.

To ensure patient safety, the APPAC III study also requires longer hospitalization compared with the standard hospital days or APPAC II trial. The APPAC III study requires covering the patient hospital costs for the three hospitalization days to ensure patient enrollment. The hospitalization costs are based on Turku University hospital prices: (38.10€ per day) for three days and for 142 patients (all together16231€).

**9. Study schedule**

APPAC II/III trials require an optimal low-dose CT protocol for the diagnosis of acute uncomplicated appendicitis. The aim of the already initiated OPTICAP trial is to optimize the low-dose CT scan and the phantom imaging protocols have already been performed and analyzed during September 2015. The clinical phase of the OPTICAP trial started in October 2015 after the acceptance notification of OPTICAP amendment for the 20.10.2015 Ethical committee meeting. The clinical phase was initiated in November 2015; first 40 patients were enrolled by the end of April. The last 20 patients were enrolled during August 2016, the data is being analyzed and the results will be available in September 2016. In October 2016 we will have an optimized low-dose CT protocol to be used in APPAC II/III trials.

The Finnish Society for Digestive Surgeons has chosen our APPAC II/III trials for creating an online research database for the society members to use as a basis for the database had to be built based on an actual trial. The costs for building such a database are 20.000€ and the costs are covered by the society. We have provided the clinical and scientific expertise for building this database, this work started already in November 2014 and the database will be used for APPAC II/III studies. The database is now finished and the programming by BCB Medical will be finished by the end of September 2016. The database will be available for use by the initiation of APPAC II trial in November 2016.

APPAC II trial protocol announcement has been already sent to the TUKIJA national committee for medical trials and their decision was to transfer the ethics committee evaluation to the local ethics committee. The APPAC II trial has been reported to and accepted by Fimea and the Eudra-CT codes are (2015-003633-10). The APPAC III trial is currently undergoing hospital pharmacy evaluations regarding the manufacturing of the trial medications. After the manufacturing plan is finished, APPAC III trial will be submitted to Fimea for final approval during the fall 2016.

Both APPAC II and III trials enroll patients from the same patient population, please see chapter 3. APPAC II trial enrollment is evaluated to last for approximately two years (until December 2018) and the primary endpoint will be analyzed at one-year follow-up at the end of 2019. APPAC III trial enrollment is evaluated for possible completion (scenarios A to C for the number of patients to be enrolled) on the 1^st^ of April 2019. For both trials, the follow-up will extend to ten years.

**10. Study hospitals and investigators**

APPAC III trial will be a national multicenter study and Turku University Hospital will be the main research center and the primary investigator will be Paulina Salminen.

Study can only be conducted at all five university hospitals (Turku, Helsinki, Tampere, Oulu and Kuopio) and one central hospital (Jyväskylä) based on the need for hospital pharmacy services for the double-blinded placebo-controlled setting.

The investigators at each research hospital: 1) Turku (Paulina Salminen, MD, PhD, Juha Grönroos, MD, PhD, Johanna Virtanen MD, PhD, Suvi Sippola, MD PhD student, Harri Marttila, MD, PhD), 2) Helsinki (Ari Leppäniemi MD, PhD, Ville Sallinen MD, PhD), 3) Tampere (Pia Nordström, MD, PhD, Johanna Laukkarinen MD, PhD, Irina Rinta-Kiikka, MD, PhD), 4) Oulu (Tero Rautio MD, PhD, Sanna Meriläinen MD, PhD) and 5) Kuopio (Tuomo Rantanen MD, PHD, Heini Savolainen, MD, PhD).

In addition, the study statistician is Saija Hurme, MSc (University of Turku) and the study health economics specialist is professor Petri Böckerman (University of Turku).

**11. References**

1.Livingston EH, Fomby TB, Woodward WA, Haley RW. Epidemiological similarities between appendicitis and diverticulitis suggesting a common underlying pathogenesis. *Archives of surgery*. 2011;146 (3):308-14.

2.Leung TT, Dixon E, Gill M et al. Bowel obstruction following appendectomy: what is the true incidence? *Ann Surg*. 2009;250 (1):51-3.

3.Margenthaler JA, Longo WE, Virgo KS et al. Risk factors for adverse outcomes after the surgical treatment of appendicitis in adults. *Ann Surg*. 2003;238 (1):59-66.

4.Fitz R. Perforating inflammation of the vermiform appendix. *Am J Med Sci*. 1886;92:321-46.

5.McBurney C. Experience with early operative interference in cases of the vermiform appendix. *NY Med J*. 1889;50:676-84.

6.Coldrey E. Treatment of Acute Appendicitis. *Br Med J*. 1956;2 (5007):1458-61.

7.Salminen P, Paajanen H, Rautio T et al. Antibiotic Therapy vs Appendectomy for Treatment of Uncomplicated Acute Appendicitis: The APPAC Randomized Clinical Trial. *Jama*. 2015;313 (23):2340-8.

8.Hansson J, Korner U, Khorram-Manesh A, Solberg A, Lundholm K. Randomized clinical trial of antibiotic therapy versus appendicectomy as primary treatment of acute appendicitis in unselected patients. *Br J Surg*. 2009;96 (5):473-81.

9.Styrud J, Eriksson S, Nilsson I et al. Appendectomy versus antibiotic treatment in acute appendicitis. a prospective multicenter randomized controlled trial. *World J Surg*. 2006;30 (6):1033-7.

10.Vons C, Barry C, Maitre S et al. Amoxicillin plus clavulanic acid versus appendicectomy for treatment of acute uncomplicated appendicitis: an open-label, non-inferiority, randomised controlled trial. *Lancet*. 2011;377 (9777):1573-9.

11.Wilms IM, de Hoog DE, de Visser DC, Janzing HM. Appendectomy versus antibiotic treatment for acute appendicitis. *Cochrane Database Syst Rev*. 2011 (11):CD008359.

12.Ansaloni L, Catena F, Coccolini F et al. Surgery versus conservative antibiotic treatment in acute appendicitis: a systematic review and meta-analysis of randomized controlled trials. *Dig Surg*. 2011;28 (3):210-21.

13.Liu K, Fogg L. Use of antibiotics alone for treatment of uncomplicated acute appendicitis: a systematic review and meta-analysis. *Surgery*. 2011;150 (4):673-83.

14.Mason RJ, Moazzez A, Sohn H, Katkhouda N. Meta-analysis of randomized trials comparing antibiotic therapy with appendectomy for acute uncomplicated (no abscess or phlegmon) appendicitis. *Surg Infect (Larchmt)*. 2012;13 (2):74-84.

15.Varadhan KK, Humes DJ, Neal KR, Lobo DN. Antibiotic therapy versus appendectomy for acute appendicitis: a meta-analysis. *World J Surg*. 2010;34 (2):199-209.

16.Varadhan KK, Neal KR, Lobo DN. Safety and efficacy of antibiotics compared with appendicectomy for treatment of uncomplicated acute appendicitis: meta-analysis of randomised controlled trials. *BMJ*. 2012;344:e2156.

17.Addiss DG, Shaffer N, Fowler BS, Tauxe RV. The epidemiology of appendicitis and appendectomy in the United States. *Am J Epidemiol*. 1990;132 (5):910-25.

18.Alvarado A. A practical score for the early diagnosis of acute appendicitis. *Ann Emerg Med*. 1986;15 (5):557-64.

19.Andersson M, Andersson RE. The appendicitis inflammatory response score: a tool for the diagnosis of acute appendicitis that outperforms the Alvarado score. *World J Surg*. 2008;32 (8):1843-9.

20.Sammalkorpi HE, Mentula P, Leppaniemi A. A new adult appendicitis score improves diagnostic accuracy of acute appendicitis--a prospective study. *BMC Gastroenterol*. 2014;14:114.

21.Berry J, Jr., Malt RA. Appendicitis near its centenary. *Ann Surg*. 1984;200 (5):567-75.

22.Korner H, Sondenaa K, Soreide JA et al. Incidence of acute nonperforated and perforated appendicitis: age-specific and sex-specific analysis. *World J Surg*. 1997;21 (3):313-7.

23.Livingston EH, Woodward WA, Sarosi GA, Haley RW. Disconnect between incidence of nonperforated and perforated appendicitis: implications for pathophysiology and management. *Ann Surg*. 2007;245 (6):886-92.

24.Lahaye MJ, Lambregts DM, Mutsaers E et al. Mandatory imaging cuts costs and reduces the rate of unnecessary surgeries in the diagnostic work-up of patients suspected of having appendicitis. *European radiology*. 2015.

25.Kim K, Kim YH, Kim SY et al. Low-dose abdominal CT for evaluating suspected appendicitis. *N Engl J Med*. 2012;366 (17):1596-605.

26.Chabok A, Pahlman L, Hjern F, Haapaniemi S, Smedh K, Group AS. Randomized clinical trial of antibiotics in acute uncomplicated diverticulitis. *Br J Surg*. 2012;99 (4):532-9.

27.Isacson D, Andreasson K, Nikberg M, Smedh K, Chabok A. No antibiotics in acute uncomplicated diverticulitis: does it work? *Scandinavian journal of gastroenterology*. 2014;49 (12):1441-6.

28.de Korte N, Kuyvenhoven JP, van der Peet DL, Felt-Bersma RJ, Cuesta MA, Stockmann HB. Mild colonic diverticulitis can be treated without antibiotics. A case-control study. *Colorectal Dis*. 2012;14 (3):325-30.

29.Isacson D, Thorisson A, Andreasson K, Nikberg M, Smedh K, Chabok A. Outpatient, non-antibiotic management in acute uncomplicated diverticulitis: a prospective study. *International journal of colorectal disease*. 2015;30 (9):1229-34.

**APPAC III trial: Statistical analysis plan for primary endpoint analyses at 10 days**

Suvi Sippola, M.D., Jussi Haijanen, M.D, Juha Grönroos, M.D., Ph.D., Saija Hurme, M.Sc., Eliisa Löyttyniemi, M.Sc., and Paulina Salminen, M.D., Ph.D., on behalf of the APPAC III study group

Version 1.2

Responsibilities:

Approved by Saija Hurme (17 Dec 2020).

Statistical analyses will be conducted by Eliisa Löyttyniemi.

SAS-programming will be done by Teemu Kemppainen.

Version 1.0->1.1 (17 Dec 2020) Time to surgery analyses added.

Version 1.1->1.2 (4 Jan2021) VAS analyses will include only time points ER (baseline), at 2-4 day follow-up, and at 10-day follow-up. Confidence intervals (95%) will be calculated for secondary outcomes as well as primary outcome for each group.

1. **Summary**

The purpose of this analysis plan is to describe the statistical data sets, procedures for conducting the analyses, and the programming details at 10-day reporting of the APPAC III trial.

1. **Trial design**

The trial has been designed as a prospective, randomized, double-blind, placebo-controlled, superiority multicenter trial to compare antibiotic therapy with placebo in the treatment of uncomplicated acute appendicitis.

1. **Registration and randomization**

Patients were randomized with a 1:1 equal allocation ratio to receive either antibiotic therapy or placebo. A safety statistician performed the randomization procedure. Randomization was performed by center using random permuted blocks. The investigators were blinded to the randomization and the randomization blocks used.

The randomization will be now open for analyses after database finalization for data collected from randomization up to the 10-day follow-up.

1. **Participants**

Patients presenting with suspected acute appendicitis will be enrolled in all five Finnish university hospitals (Turku, Oulu, Helsinki, Tampere, and Kuopio).

1. **Sample size calculations**

Sample size calculations were based on one-sided Pearson’s χ^2^ -test for two proportions. Sample size was calculated from an estimated success rate of 94% during the primary hospitalization in antibiotic group^1^. A decrease of 15 percentage points in success rate is considered clinically important difference leading to estimated 79% success rate in placebo group. We estimated that to detect a 15 percentage point difference (antibiotics – placebo) between the groups with a power of 0.8 (1-β) and one-sided significance level (α) of 0.05, 64 patients per group is needed. Before the actual trial initiation, 3-5 pilot patients were be recruited in order to finalize the hospital pharmacy procedures and drug delivery in clinical practice. Neither the study protocol nor the hospital pharmacy procedures were in any way changed based on the pilot patients, i.e. all patients underwent the same study protocol and procedure. The pilot patients were thus included in the actual study patients in the final analysis. The trial registration (Eudra-CT 2015-003634-26) was performed prior to the pilot patient registration and stayed unchanged; the trial was also registered to clinicaltrials.gov (NCT 03234296). Based on the pilot study enrollment speed and the recognized challenges in conducting trial, in real-life emergency setting we needed to take into account the anticipated enrollment delays to assure the completion of this trial within reasonable time. These challenges consisting of emergency surgery patient enrollment dependent on hospital pharmacy services available only during standard working hours, the requirement for senior surgeon enrollment and the discrepancy between the admission hours of appendicitis patients and hospital pharmacy working hours, mandated us to create three scenarios for study power analysis and the number of patients to be enrolled.

In scenario A, a decrease of 15 percentage points in success rate was considered clinically important difference leading to estimated 79% success rate in placebo group. We estimated that to detect a 15-percentage points difference (antibiotics – placebo) between groups 64 patients per group is needed. With an estimated dropout rate of 10% total of 142 patients, 71 patients per group will be enrolled in the study. In scenario B, clinically important difference was 20 percentage points, estimated success rate in placebo group was 74% and to detect this difference 41 patients per group were needed. With an estimated dropout rate of 10% total of 92 patients, 46 patients per group will be enrolled in the study. In scenario C, clinically important difference was 25 percentage points, estimated success rate in placebo group was 69% and to detect this difference 29 patients per group is needed. With an estimated dropout rate of 10% total of 64 patients, 32 patients per group will be enrolled in the study. Targeted minimum sample size per study hospital will be 10 patients. One-sided test will be used as our hypothesis is that antibiotic treatment is more effective treatment than placebo. Sample size calculations were performed using Power procedure in SAS System for Windows, Version 9.4 (SAS Institute Inc., Cary, NC).

1. **Statistical hypothesis**

The primary objective of the study is to compare the success of randomized treatment comparing antibiotic therapy and placebo at ten days after initiation of the randomized treatment. Superiority of antibiotic group vs. placebo group will be tested using following statistical hypothesis (typo in study protocol has been corrected in null hypothesis below):

H_0_: p_1_ ≤ p_2_

H_1_: p_1_ > p_2_

where p_1_ is success of treatment proportion of antibiotic group and p_2_ for placebo group.

1. **Statistical analyses**
   1. General considerations

Categorical variables of the study will be characterized by treatment using frequencies and percentages and for continuous variables with means and standard deviations (SD) or medians with range and/or 25^th^ and 75^th^ percentiles will be used. Age will be summarized with mean, SD and range.

The assumptions of parametric tests will be checked using studentized residuals for justification of the analyses.

P-values less than 0.05 will be considered statistically significant (one-sided in primary analyses, two-sided otherwise). Confidence intervals of 95% will be calculated. Statistical analyses will be performed using SAS System for Windows, Version 9.4 or later (SAS Institute Inc., Cary, NC).

- 1. Analysis population

The main analyses will be based on the intention-to-treat (ITT) principle (all randomized excluding possible erroneously randomized patients with CT diagnosis of complicated appendicitis and early drop outs). Early drop out will be defined as a patient who withdraws consent early without receiving actual allocated treatment. The subjects with missing data will automatically be excluded from the analyses of the variables in concern.

- 1. Baseline comparison

Baseline characteristics will be summarized for whole study population and separately for each treatment group. Baseline comparison will be performed with Fisher’s exact test, two-sample t-test, or Wilcoxon rank sum test. Also linear model, where study site is considered as random effect, can be considered for normally distributed data. For categorical data e.g. binomial generalized linear models, a log-binomial model can be performed.

- 1. Primary outcome

The treatment success is defined as the resolution of acute appendicitis with study treatment resulting in discharge from the hospital without the need for surgical intervention and treatment efficacy will be evaluated at ten days after initiation of the randomized treatment. The patients undergoing appendectomy during the initial hospitalization will also be analyzed to assess the appendicitis status at surgery and to evaluate patients with primary treatment failure. To validate this accuracy of the differential diagnosis between uncomplicated and complicated acute appendicitis, all patients will be assessed at all time points either using clinical data and CT findings for patients with uncomplicated acute appendicitis treated with antibiotics, or additionally for patients undergoing appendectomy also by surgical and histopathological findings. All clinical diagnoses were assessed in a blinded manner by two PhD students not being aware of the other evaluation. In cases of disagreement, the clinical diagnosis was reviewed by the study PI.

Difference in treatment success between antibiotic and placebo group will be tested using Fisher´s one-sided test (at 0.05 level). The two-sided 90% confidence interval (CI) for proportion difference will be calculated to estimate the treatment difference.

- 1. Secondary outcome

Secondary endpoints **at 10-day analyses** include

- post-intervention adverse events/complications (Clavien-Dindo classification),
- duration of hospital stay,
- VAS scores (in the ER (baseline)*, daily in the ward, at discharge, at 2-4 day follow-up, and at 10-day follow-up*). Time points marked with * will be included in repeated measures analyses described below.
- sick leave
- time to appendectomy (time from ER to surgery)

Other secondary endpoint variables analyzed

- leucocyte counts, CRP (in the ER, daily in the ward, at discharge, 2-4, and/or 10 days after discharge
- temperature recorded in the ER and upon admission
- additional CT findings outside specific CT diagnosis criteria (for example appendiceal diameter)

All recorded pre-intervention variables will be used to evaluate potential prognostic factors for primary treatment failure of non-operative treatment. The secondary outcomes (adverse events/complications, duration of hospital stay, sick leave) will be analyzed using chi-square test/Fisher’s exact test, independent samples t-test or Mann-Whitney U-test. The assumptions of tests will be checked for justification of the analyses. For the secondary outcomes two-sided p-values will be used. The study site differences will be evaluated in statistical models and if major differences are detected more complicated statistical models will be used in the analyses of primary and secondary outcomes.

Time to appendectomy will be calculated from ER to surgery or time to ER to 10 day (if surgery not performed, censored cases) and then analyzed using log-rank test and Kaplan-Meier curve will be drawn to illustrate the phenomenon. In addition, Cox’s proportional hazard model can be build up to study possible effect of prognostic factors (see list of potential prognostic factors below). Assumptions for proportional hazard function will be checked.

Change analyses for VAS, CRP and leucocyte count

If assumptions for parametric analyses are met, mean change over time will be analyzed using linear mixed modelling approach for repeated measures. Then time point will be considered as within-factor and treatment group as between factor. Time point x group interaction will describe whether mean changes over time are different between the treatment groups. Study center will be handled as random effect. Covariance structure of unstructured (UN) and compound symmetry (CS) will be tested. Differences (together with 95% CI) between the groups will be also estimated at each time point, likewise within group changes. Square root transformation, as well as logarithmic transformation can be performed before analyses, if needed.

Prognostic factor modelling

Possible prognostic factor evaluation for primary treatment failure (i.e. complicated acute appendicitis at surgery during the initial hospitalization) will be evaluated.

Studied potential prognostic factors:

- age
- gender
- body mass index
- VAS score in the ER
- leukocyte count
- C-reactive protein
- body temperature in the ER
- appendiceal diameter in CT
- minor fluid collection around appendix in CT
- edema around appendix in CT
- symptom duration prior to hospitalization

In addition, randomization group (fixed) and study center (random) effects will be included in the models as study design factors.

Analyses were performed with binomial generalized linear models, i.e. log-binomial model (SAS/PROC GLIMMIX).

First, univariate approach will be executed i.e. one prognostic factor at the time will be evaluated, always including study center and randomization group as study design factors. All numerical factors will be handled as continuous in the models at this stage. In addition, interaction of group x factor will be included in the model. If this interaction is not significant, it will be removed from the model. Consequently the multivariable model will be constructed including both study design factors and all significant prognostic factors from univariate models.

Finally, optimal and clinically meaningful cut off values for significant factors will be evaluated to get estimates from the model to aid in clinical interpretation.

References

1. Salminen P, Paajanen H, Rautio T et al. Antibiotic Therapy vs Appendectomy for Treatment of Uncomplicated Acute Appendicitis: The APPAC Randomized Clinical Trial. *Jama*. 2015;313 (23):2340-8.
